# Microglia: An Active Player in the Regulation of Synaptic Activity

**DOI:** 10.1155/2013/627325

**Published:** 2013-11-03

**Authors:** Kyungmin Ji, Jeremy Miyauchi, Stella E. Tsirka

**Affiliations:** Department of Pharmacological Sciences, Stony Brook University, Stony Brook, NY 11794-8651, USA

## Abstract

Synaptic plasticity is critical for elaboration and adaptation in the developing and developed brain. It is well established that astrocytes play an important role in the maintenance of what has been dubbed “the tripartite synapse”. Increasing evidence shows that a fourth cell type, microglia, is critical to this maintenance as well. Microglia are the resident macrophages of the central nervous system (CNS). Because of their well-characterized inflammatory functions, research has primarily focused on their innate immune properties. The role of microglia in the maintenance of synapses in development and in homeostasis is not as well defined. A number of significant findings have shed light on the critical role of microglia at the synapse. It is becoming increasingly clear that microglia play a seminal role in proper synaptic development and elimination.

## 1. Microglia

Microglia constitute approximately 10% of the cells in the CNS. They have been traditionally thought to function as the immunocompetent cells of the brain and spinal cord [1] and to be the sensors of injury and infection in the tissue [2, 3]. They derive from primitive c-kit(+) erythromyeloid precursors from the yolk sac [4–6], migrate into the brain during the period of early embryonic development prior to the formation of the blood-brain barrier (BBB), and remain there once the BBB is formed [7]. It is notable that this population is self-sustaining, and peripheral macrophages only contribute to this population in disease states, in which the BBB becomes compromised [8].

Within the brain, microglia have been described to have the ability to detect and mount an inflammatory response to various insults. Sensing neuronal injury has been ascribed primarily to purinergic and chemokine receptors on the surface of microglia, as they monitor the levels of extracellular ATP and secreted chemokines, respectively [9]. Their reaction to neuronal injury is accomplished as they undergo a process collectively called “activation.” Activation consists of several biological events that include migration to the site of injury, local proliferation, a change in morphology and gene expression, antigen presentation, and phagocytosis of dead cells and cell debris [10, 11]. During activation, some of the changes in gene expression involve the secretion of cytokines and chemokines, which modulate the CNS environment and regulate the state of inflammation. Inflammation in turn affects the progression of neuronal death after CNS insult. Microglia can secrete both proinflammatory mediators, such as tumor-necrosis-factor- (TNF-) *α* [12, 13] or interleukin- (IL-) 1*β*, nitric oxide (NO) [14, 15], and glutamate [16], and anti-inflammatory effectors, such as IL-4 and IL-13, which can enhance neuronal survival [17, 18]. 

Depending on the predominance of factors secreted, microglia have classically been characterized, similarly to macrophages, as M1 (proinflammatory) or M2 (anti-inflammatory) cells [19]. The M1 and M2 distinctions serve to separate activated microglia into the two broad categories. It is argued, however, that no specific marker designates a microglial response as definitively M1 or M2. Moreover, microglia differentially express pro- and anti-inflammatory markers making the M1 and M2 phenotypes the extremes of the categories, respectively, with variable phenotypes seen in between the two [8]. With that in mind, M1 microglia have been associated with neurotoxic and neurodegenerative outcomes, as they are observed in a variety of chronic neurodegenerative diseases, such as Alzheimer's [20], end-stage amyotrophic lateral sclerosis (ALS) [21], and multiple sclerosis (MS) [22]. Stroke [23] and traumatic brain injury [24] show a characteristic accumulation of M1 microglia as well. A shift to an M2 phenotype of activated microglia has been correlated with neuroprotection, recovery, and repair in various disease settings [25–27]. 

## 2. Microglia as Regulators of Neuronal Function and Plasticity

Until recently, initial studies to understand neuronal-microglial interactions had described that a variety of neuroactive substances, such as NO [29] and TNF-*α* [30], have potent effects on neuronal function, in particular, synaptic plasticity. However, the cellular origin of these molecules had not been attributed to microglia but, rather, to astrocytes. The focus was maintained on the effect that inflammatory processes have on synaptic plasticity. In neuroinflammatory diseases, such as experimental autoimmune encephalomyelitis (EAE), a rodent model of MS, alterations in synaptic plasticity have been noted. Specifically, in the hippocampus of diseased animals, there is greater induction of long-term potentiation (LTP), an electrophysiological measurement that relates to the connectivity and strength of synapses. This change in LTP was attributed to the secretion of IL1*β* from the accumulated microglial cells [31]. Bacterial lipopolysaccharide (LPS) strongly upregulates IL1*β* secretion by macrophages. 

It is notable, however, that prolonged exposure to inflammatory cytokines can result in priming or sensitization of microglia so they more readily adopt an M2, rather than M1, phenotype in response to inflammation. This is quite the opposite response to that in acute exposure [32]. As such, chronic inflammation can be induced by LPS infusion and has been reported to attenuate LTP in the dentate gyrus (DG) of the hippocampus. This is accompanied by the loss of pyramidal neurons [33]. Similarly, using LPS infusion, Min et al. found that LTP, dependent on either NMDA receptors (NMDAR) or on voltage-dependent calcium channels, was impaired [34]. Further work is necessary to elucidate the specific mechanism causing these phenomena.

The cytokine, TNF-*α*, increases the surface expression of AMPA receptors in neuronal cultures, which is accompanied by the enhancement of synaptic strength [30]. In a model of neuropathic pain in the C fibers of the dorsal horn of the spinal cord, inhibition of microglial activation by minocycline resulted in the induction of long-term depression (LTD) rather than LTP. This change in plasticity was found to involve Src family kinases and to be mediated partially by TNF-*α* [35]. Some studies have found that microglial activation, when both genetically and pharmacologically induced, results in an increase of AMPAR/NMDAR ratio and an enhanced ratio of AMPAR- over NMDAR-mediated currents [36]. These studies demonstrated that when microglia were activated under pathological inflammatory conditions, they caused synaptic alterations via secretory mediators. The precise role of microglia on synaptic activity in the normal brain remained unclear. 

Imaging studies have shown that microglia extend and retract their processes continuously to survey their local environment in the healthy brain [37, 38]. Moreover, interactions between microglia and neuronal synapses in the visual cortex have been directly visualized by electron microscopy (EM) and by in vivo two-photon microscopy. The availability of visual stimuli resulted in enhancement of the duration of these contacts and the preservation of the synapse [39]. These intriguing imaging observations first indicated the possibility that microglia could modulate neuronal functions by direct physical contacts. On the other hand, Wake et al. demonstrated that, under conditions of prolonged ischemia, contact time between microglial processes and synapses increased, associated with a greater chance for elimination of presynaptic boutons [40]. It is likely that the mechanism of synaptic pruning is modulated by a distinct molecular mechanism in each of these states and not simply by the longevity of contact.

## 3. Mechanisms Governing the Interactions of Neurons and Microglia

Paolicelli et al. [41] explored whether there is a functional role for microglial interactions with synaptic structures during the development of the postnatal brain, using imaging and electrophysiological approaches. They used a transgenic mouse line expressing GFP in microglia, under the control of the chemokine receptor CX3CR1 promoter. Thus, they were able to label and visualize microglia as well as manipulate them. The authors found that the number of synaptic elements and dendritic spines expressing the postsynaptic marker PSD95 in Cx3cr1^GFP/+^ mice was about 3-fold higher than that in mice deficient in CX3CR1  (Cx3cr1^KO/KO^). Their result provided some insight into potential roles of microglia in synapse maturation, along with the possibility that this may be a direct CX3CR1-mediated event [42]. These microglial properties, thus, extend beyond immune surveillance and indicate modulatory roles during normal brain development. 

Schafer et al. [43] provided direct evidence, via confocal microscopy and electron microscopy, that microglia phagocytose synapses in the dorsal lateral geniculate nucleus (dLGN) as well. The authors proposed that the classical complement cascade, which includes members C1q and C3, was a potential molecular pathway of microglia-synapse interactions in postnatal brain development. According to the experimental data, the synapses that were tagged with C1q and C3 were phagocytosed by microglia that expressed complement receptor CR3. In mice deficient in the receptor or the ligand, higher numbers of synaptic inputs were observed. Moreover, these animals showed deficits in their ability to segregate the territories of each eye. Therefore, the microglia-mediated engulfment was important to drive synaptic stripping during normal development. Together, these observations reveal that complement-mediated phagocytic activity of microglia is crucial in microglia-synapse interactions during normal brain development.

**Figure 1:**
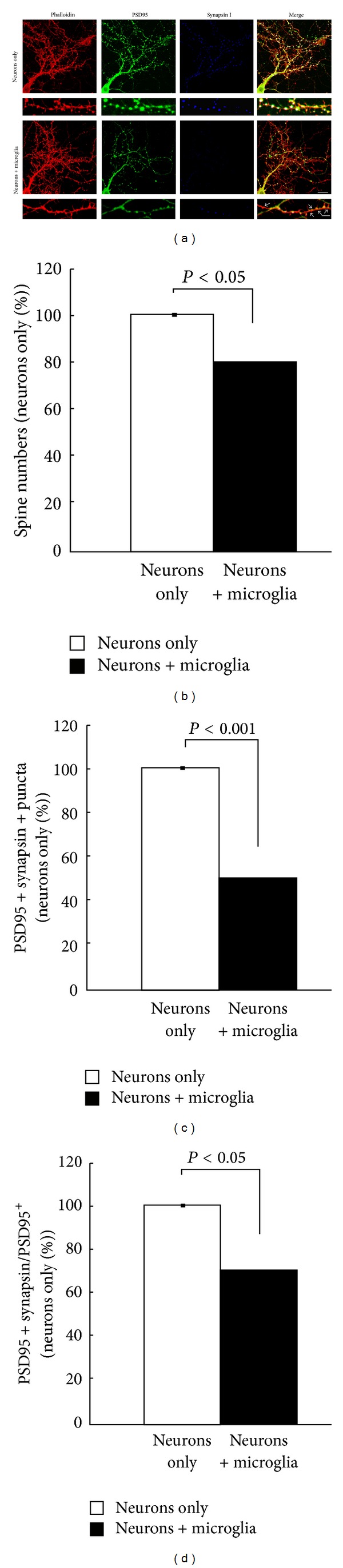
Microglia alter the synaptic density of hippocampal neurons. Hippocampal neurons with or without microglia were stained with PSD95 (green), synapsin I (blue), and phalloidin (red) (a). The smaller boxes show magnified images. Arrows depict PSD95^+^ synapsin 1^−^ puncta. Scale bars: 20 *μ*m (upper panel); 5 *μ*m (lower panel). Quantification of spine numbers (b), PSD95^+^synapsin 1^+^ puncta (c), and PSD95^+^ synapsin 1^+^ puncta in total PSD95^+^ puncta (d) in neurons cultured with or without microglia. Values are presented as mean ± SEM and expressed as a percent of the neurons-only control sample (adapted from [28]).

To further address whether microglia contribute to synaptic activity in the normal young adult brain, Ji et al. [28] used an electrophysiological approach in organotypic hippocampal brain slices and primary neuronal cultures. In this system, they manipulated the presence of microglia by either depleting them using clodronate, replenishing them in previously depleted cultures, or by using cocultures of neurons and microglia. The absence of microglia resulted in a robust increase of synaptic frequencies known as spontaneous and miniature excitatory postsynaptic currents (sEPSC and mEPSC) from the CA1 region of the hippocampus. This increase was subsequently reversed when microglia were replenished in the organotypic slices. In the complementary approach, the addition of microglia to neuronal cultures decreased the synaptic activity measured compared to cultures of neurons alone. The change in synaptic activity coincided with changes in synaptic numbers, which suggested that microglia could participate in the control of synaptic activity by regulating synaptic numbers (Figure 1). As shown in previous reports [43], they also observed that the phagocytic activity of microglia drove synapse elimination when microglia were coincubated with neurons. This could be one mechanism by which synapse numbers are regulated in the normal brain; however, it is still undefined whether synaptic pruning and phagocytic engulfment by microglia occur via a universal mechanism under normal and pathological conditions.

**Figure 2:**
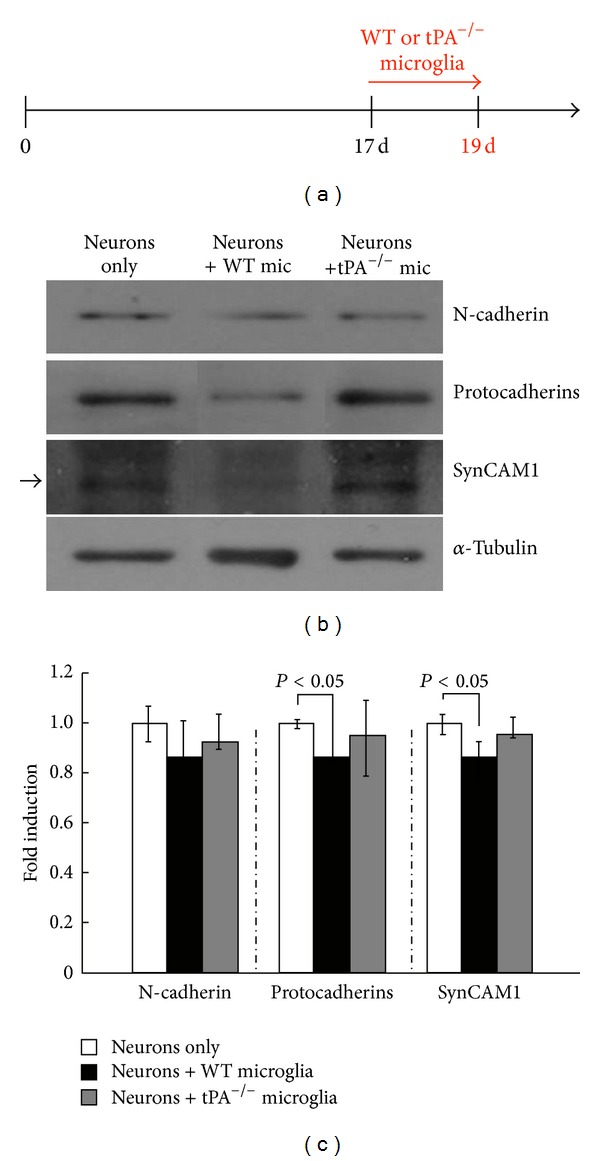
Microglial tPA deficiency preserves the levels of synaptic adhesion molecules. (a) Hippocampal neurons at 19 DIV were cocultured with microglia for 2 days. (b) The western blot analysis of the levels of N-cadherin, pan-*γ*-protocadherin, and SynCAM-1 in neurons in the absence or presence of microglia from wild-type (WT) or tPAKO(tPA^−/−^) mice. *α*-Tubulin was used as a loading control. (c) Quantification was performed using the ImageJ software and normalized against *α*-tubulin (*n* = 4). *P < 0.05 compared to neurons alone.

A proposed mechanism by which microglia could regulate synaptic activity was suggested by the same study [28]. Overall expression of synaptic adhesion molecules, such as protocadherin and SynCAM1, which determine synapse remodeling, stability, and synaptic activity, was decreased in neurons incubated with microglia compared to neurons alone (Figure 1). The decreased levels of the synaptic adhesion molecules were recovered to wild-type levels when neurons were incubated with microglia deficient in the serine protease tissue plasminogen activator (tPA) (Figure 2), potentially implicating serine proteolytic functions in the stability of these proteins. 

Structural changes at the synapse are closely associated with synaptic stability. In particular, numerous synaptic adhesion molecules, such as classic cadherins (E-cadherin and N-cadherin), protocadherins, and NCAM, have been studied in modulating structural and functional synaptic plasticity. Hippocampal slices pretreated with antibodies against the extracellular domain of N- and E-cadherins or with antagonistic peptides that inhibit cadherin dimerization exhibit a significantly reduced LTP [44]. Moreover, expression of mutant N-cadherin or short hairpin RNA-mediated knockdown of N-cadherin prevents LTP-induced long-term stabilization of synapses [45]. Additionally, Yamagata et al. showed that blocking antibodies to protocadherins or NCAM in hippocampal slices diminished synaptic transmission and LTP induction [46]. 

Proteases in a synaptic microenvironment are important in the regulation of dynamic changes in the adhesion molecules associated with synaptic plasticity [47]. Proteases, such as matrix metalloproteinases (MMPs) and tPA, secreted from neurons, astrocytes, or microglia under basal or pathological conditions of the CNS have been associated with the targeted degradation or proteolytic processing of extracellular matrix (ECM) and cell adhesion molecules on the cell surface and at the synapse [48–55]. In particular, application of tPA or MMP-9 was shown to be involved specifically in the production of LTP and synaptic growth. Emerging evidence has shown that application of MMP-9 or tPA decreases the levels of N-cadherin and diminishes synaptic transmission [56]. Moreover, tPA regulates MMP activity [57], which leads to the regulation of synaptic plasticity. It is possible that proteases secreted from microglia could regulate synaptic activity by remodeling the ECM which is known to affect synaptic connectivity [58]. 

## 4. Connexins and Large Pore Channels

One way of communication among microglia is through connexins and large pore channels. Connexins (Cx) are proteins found in gap junctions, connecting adjacent cells. Each of the connected cells provides an array of Cx isoforms, which form oligomers containing 6 of these Cx proteins. This complex is called a connexon and constitutes a hemichannel [59]. The most common isoforms in mammals are Cx36, Cx43, and Cx45. Connexins are traditionally described as being expressed in astrocytes and in neurons. Cx36 and Cx43 have been reported to be expressed in microglia [60, 61], where they are thought to be involved in the local release of proinflammatory cytokines (TNF-*α* and IL1*β*) [62] and metabolites. During inflammatory events, the expression of Cx43 was shown to increase. This increase results in the formation of a functional syncytium among microglial cells, confirmed by the diffusion of the fluorescent dye, Lucifer yellow. However, the syncytium neither forms in nonactivated microglia [63], nor happens if the gap junction formation is inhibited by inhibitors of Cx43, indicating the involvement of Cx43 in the process. Cx36 remains active in resting microglia and does not become upregulated during microglial activation. 

In disease settings, it has been reported that blocking Cx hemichannels resulted in the blockade of the microglial release of glutamate [64], which led to the subsequent exaggerated activation of neurons (excitotoxicity). In a model of spinal cord injury (partial cord transection), inhibition of Cx resulted in improved functional recovery [65]. 

Similar to Cx proteins, large pore channels are formed in microglia and consist primarily of pannexins and P2X channels. They are purinergic and activated by extracellular ATP. Among them, P2X_4_ is the channel that becomes primarily upregulated in activated microglia [63]. In a recent report, Li et al. state that in the optic tectum of larval zebrafish, neuronal activity drives the activation of pannexin-1 hemichannels. These can then “steer” the processes of resting microglia and facilitate their contact with highly active neurons [66]. In turn, when resting microglia are in contact with neurons or neurites, a decrease in both spontaneous and visually evoked neuronal activities is observed, specifically for the neurons contacted.

These results indicate that connexins and large pore channels could constitute one way by which microglia interact directly with neurons, especially during neuronal insult and inflammation, and could directly affect neuronal activity and survival.

## 5. Direct Modulation of Neurotransmitter Release and Homeostasis

As mentioned above, microglia can generate neurotransmitters, primarily glutamate. They also respond to changes in neurotransmitters by changing morphology and the motility of their processes. Such responses have been documented both for glutamatergic but also for GABAergic transmission [67]. Application of the glutamate receptor inhibitors NBQX and GYKI, as well as the GABAergic signaling inhibitor bicuculline, has been shown to decrease microglial process motility [68]. Although there is debate on whether microglia express glutamate receptors [68], the presence of GABA_A_ receptors on the surface of human microglia has been documented [69]. However, there is no concrete evidence that microglia respond in an obvious way to direct application of agonists of glutamate or GABA receptors, that is, in a pure microglial cell culture. Rather, they seem to respond indirectly to such application, on a slice or tissue, suggesting that these agonists potentially have indirect effects on the cells. These indirect effects have been postulated to be mediated through ATP's effect on purinergic receptors since they are expressed on the surface of the cells [37]. Although the source of the ATP release is not entirely determined, the most likely mechanism involves release through neuronal pannexin channels [70].

In models of disease, specifically in the EAE model of MS, the presence and accumulation of activated microglia have been correlated with decreases in the Purkinje cell survival, connectivity in the cerebella of the EAE animals, and attenuation of GABAergic transmission [71]. This has also been observed in the EAE striatum [72] and hippocampus, where a decrease in GABAergic interneurons was also noted, accompanied by induction of LTP [31]. These results suggest that, in this context, microglia may be direct regulators of the numbers of GABAergic neurons and the subsequent attenuation of GABAergic inhibitory transmission.

## 6. Ectosome and Lipid Signaling

A new way of communication in the CNS has been described involving the release of microvesicles, also referred to as *shed vesicles* or *ectosomes*, from the plasma membrane [73]. These materials were originally thought to be inert but have been recently recognized as critical in mediating cell-to-cell communication. The vesicles contain lipids, cell surface proteins, and material from the cytoplasm or nucleus of the cell [74]. The vesicles are recognized by the recipient cell through the presence of phosphatidylserine on their surface [75] and interact with the relevant receptors. They can also directly fuse with the recipient cell. 

On the surface of microglia, P2X_7_ receptors, which respond to the release of ATP, mediate the shedding of ectosomes [76]. This process is triggered by the activity of acid sphingomyelinase and involves the activation of the effector protein p38. Although this is not a mechanism exclusive to microglia (as astrocytes also have been shown to express the P2X_7_ receptors), microglia constitute a significant source of these shed vesicles. 

Signaling through these microvesicles has been reported in different systems. One of the factors thought to contribute to such signaling is Annexin A2 [77], a protein expressed by microglia that affects their activation [78, 79]. Annexin A2 has been shown in different systems to affect neuronal ion channels and neuronal functioning [80, 81], either directly or through its interaction with p11 [82].

In a recent report, it was noted that microvesicles derived from microglia were able to increase the frequency and amplitude of EPSCs [74]. This effect required interaction between microglia and neuronal cells and did not involve secretion of cytokines. It did involve, however, an increase in the metabolism of sphingolipids in neuronal cells. This resulted in an acute increase in excitatory neurotransmitter release. Although in a more chronic exposure to these shed particles the release of cytokines as regulators of neuronal activity cannot be excluded, these data provide another possible pathway by which microglia affect neuronal activity.

## 7. Conclusions

**Figure 3:**
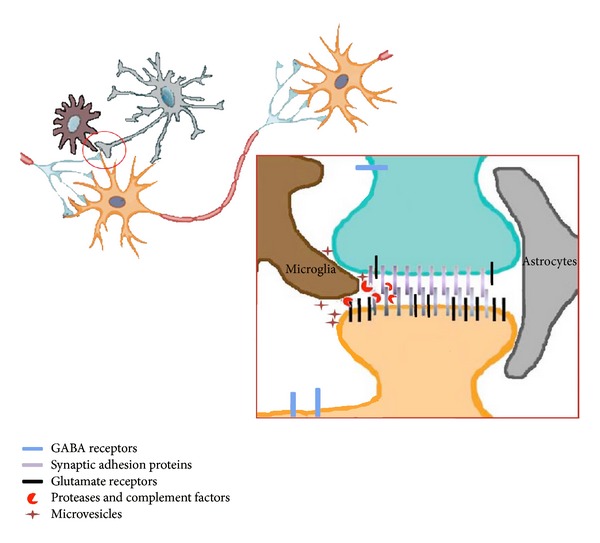
Schematic depicting some of the potential mechanisms through which microglia can affect neuronal activity. Potential interactions between neurons, astrocytes, and microglia at the synapse. The area depicted by the red circle is magnified in inset. Inset: microglial processes in proximity to neuronal synapses can modify neuronal activity via multiple potential pathways. They can secrete proteases to modulate the stability of synaptic adhesion molecules (which in turn influences synaptic transmission) or remove complement-tagged structures. They can release ectosomes that directly interact with the neuronal membranes and initiate signaling cascades. They can also affect directly glutamatergic or GABAergic transmission.

Given the evidence from imaging, cellular, and electrophysiological approaches, the physical proximity between neurons and microglia seems to result in synaptic maturation and synaptic activity (Figure 3). Several different mechanisms, either involving direct contact and interaction between the two cell types, or mediated through chemical ligands and effectors [9], are described as potential regulators of these microglial functions. The findings indicate that microglia affect both the maturation of the CNS during development and the acute and dynamic regulation of neuronal activity in the mature, healthy, or unhealthy CNS and suggest that they are active contributors to a potential quad-partite synapse [83].
